# Crystal structure of 5-[(benzo­yloxy)meth­yl]-5,6-dihy­droxy-4-oxo­cyclo­hex-2-en-1-yl benzoate

**DOI:** 10.1107/S2056989020007793

**Published:** 2020-06-19

**Authors:** Theerachart Leepasert, Patchreenart Saparpakorn, Kittipong Chainok, Tanwawan Duangthongyou

**Affiliations:** a Department of Chemistry, Faculty of Science, Kasetsart University, Bangkok 10900, Thailand; b Thammasat University Research Unit in Multifunctional Crystalline Materials and Applications (TU-MCMA), Faculty of Science and Technology, Thammasat University, Khlong Luang, Pathum Thani, 12121, Thailand

**Keywords:** crystal structure, *Pipers griffithii*, zeylenone, hydrogen bonds

## Abstract

The crystal structure of the natural product zeylenone, C_21_H_18_O_7_, was confirmed by single-crystal X-ray diffraction. The crystal structure has three chiral centers at positions C1, C5 and C6 of the cyclo­hexa­none ring, but the absolute configuration could not be determined reliably. The methyl benzoate and benzo­yloxy substituents at positions C1 and C5 of the cyclo­hexenone ring are on the same side of the ring with the dihedral angle between their mean planes being 16.25 (10)*°*. These rings are almost perpendicular to the cyclo­hexenone ring. The benzoate groups and two hydroxyl groups on the cyclo­hexenone ring form strong hydrogen bonds to consolidate the crystal structure. In addition, weak C—H⋯O hydrogen bonds also contribute to the packing of the structure.

## Abstract 2

The crystal structure 5-[(benzo­yloxy)meth­yl]-5,6-dihy­droxy-4-oxo­cyclo­hex-2-en-1-yl benzoate a natural product from *Pipers griffithii* leaves known as zeylenone has been determined.

## Chemical context   

Zeylenone is a naturally occurring polyoxygenated cyclo­hexene derived from the shikimate pathway. It has been found in a few plant families such as *Piperaceae* and *Annona­ceae*. The biological activity of zeylenone was reported as inducing apoptosis in the mitochondria of gastric cancer cells (Yang *et al.*, 2018 ▸) and cervical carcinoma cells (Zhang *et al.*, 2017 ▸). The absolute configuration of natural zeylenone was determined by CD spectroscopy to be (−)-zeylenone (Takeuchi *et al.*, 2001 ▸).
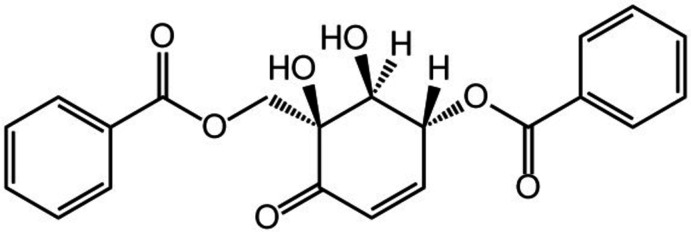


## Structural commentary   

**Figure 1:**
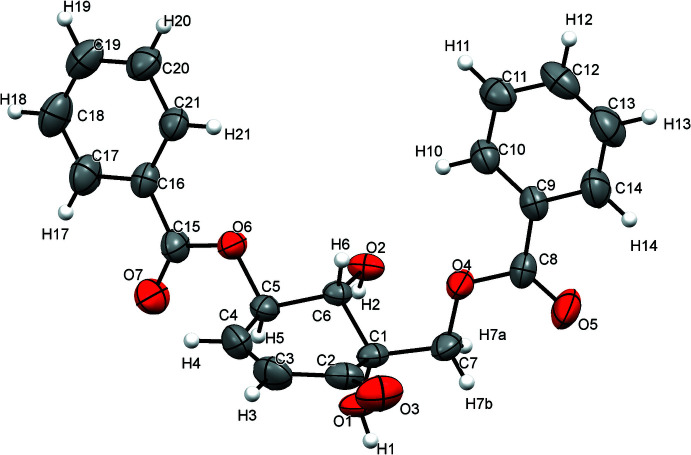
The mol­ecular structure of compound (I) with the atom labelling and 50% probability displacement ellipsoids.

The mol­ecular structure of the title compound (I) is shown in Fig. 1 ▸. It has three chiral centers at positions C1, C5 and C6 of the cyclo­hexa­none ring. However, the absolute configuration (probably 1*S*, 5*R* and 6*S*) could not be deduced from the X-ray data because of the large standard deviation of the Flack parameter [0.0 (3)]. The two main substituents are methyl benzoate and benzo­yloxy at positions C1 and C5, and positioned at the same side of the cyclo­hexenone ring. The dihedral angle between the methyl benzoate and benzo­yloxy mean planes is 16.24 (10)°, indicating that the rings are almost coplanar. The dihedral angle between the cyclo­hexenone ring and the methyl benzoate and benzo­yloxy rings are 74.92 (9) and 69.23 (10)°, respectively, indicating that the aromatic and cyclo­hexenone rings are almost perpendicular. The conformation of the cyclo­hexenone ring, the core structure of (−)-zeylenone, is described as a half-chair based on the torsion angles H4—C4—C3—C2 [−178.7 (3)°, almost planar] and C5—C6—C1—C2 [−60.65 (16)°, perfectly staggered] and the puckering parameters [*Q* = 0.4989 (17) Å, θ = 130.8 (2)° and Φ = 143.9 (3)°].

## Supra­molecular features   

**Figure 2:**
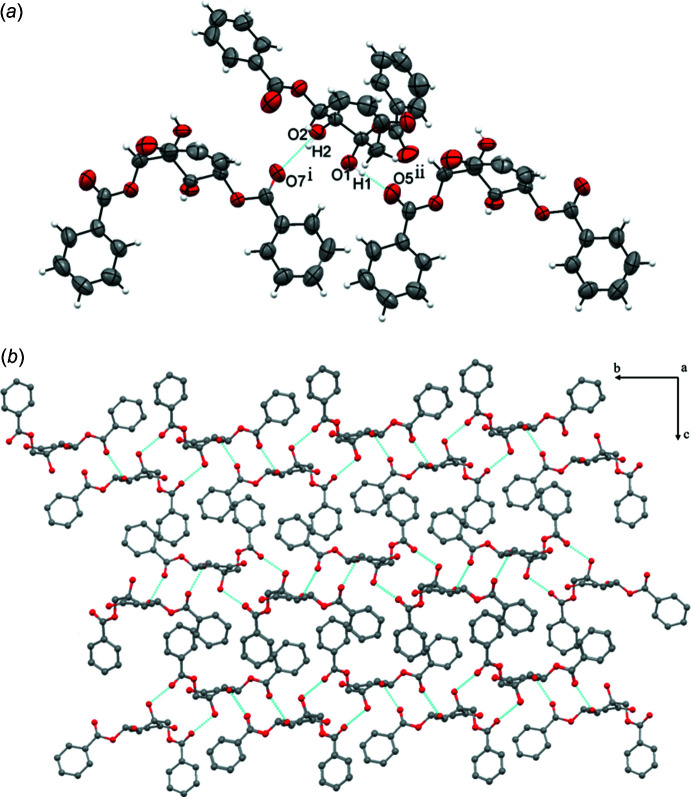
(*a*) O—H⋯O hydrogen bond formation in (I) and (*b*) the crystal packing viewed along the *a* axis. Hydrogen bonds are shown as dashed lines.

**Figure 3:**
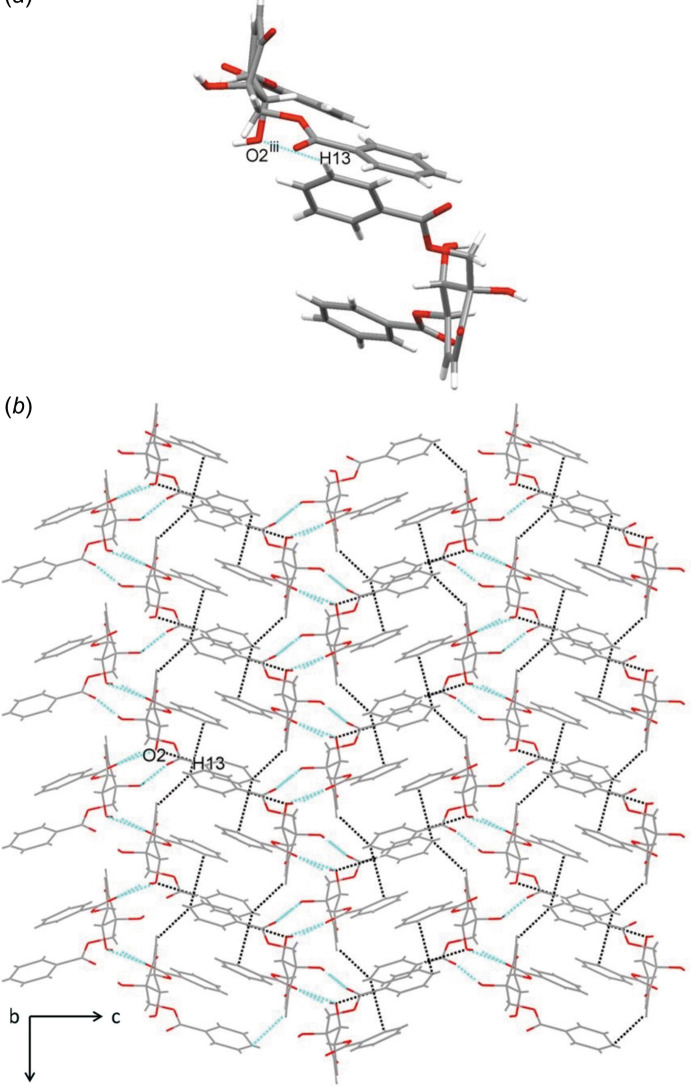
(*a*) C—H⋯O hydrogen bonds and (*b*) the crystal packing viewed along the *b* axis. Blue dashed lines represent O—H⋯O hydrogen bonds and black dashed lines represent the C—H⋯O and the weak π–π inter­actions.

The crystal packing is characterized by both strong and weak hydrogen bonds and also by partial π–π inter­actions. The strong hydrogen bonds are formed between hydroxyl groups on the cyclo­hexenone ring and the uncoordinated oxygen atom of methyl benzoate and benzo­yloxy substituents (O2—H2⋯O7^i^ and O1—H1⋯O5^ii^, Fig. 2 ▸ *a*, Table 1 ▸). These inter­actions form a layer parallel to the *bc* plane (Fig. 2 ▸ *b*). In addition, the crystal packing features weak C—H⋯O hydrogen-bonding inter­actions (C13—H13⋯O2^iii^, Table 1 ▸) and contacts between the aromatic rings [the shortest centroid–centroid distance between phenyl rings is 4.641 (2) Å], as shown in Fig. 3 ▸.

## Computational calculations   

The structure of the title compound was optimized using density functional theory (DFT) calculations at the M062X/6-31G(d) level using *GAUSSIAN 09* (Frisch *et al.*, 2016 ▸). The optimized structure was then used for the analysis of the highest occupied mol­ecular orbital (HOMO) and the lowest unoccupied mol­ecular orbital (LUMO) using the same level of theory in order to determine the reactivity of the compound *via* the energy gap.

**Figure 4:**
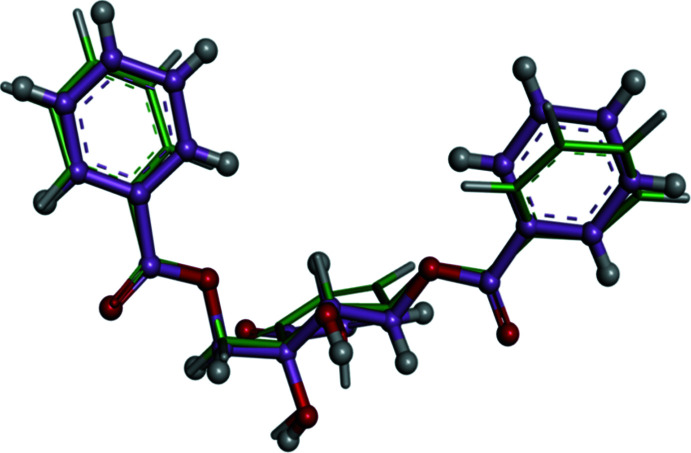
Superposition of the experimental (ball-and-stick model) and optimized (stick model) structures.

The DFT-optimized geometry was compared with the geometry obtained from the crystal structure using the mol­ecular overlay module based on 50% steric and 50% electrostatic similarities in the *Discovery Studio* visualizer (Dassault, 2018 ▸), as shown in Fig. 4 ▸. The overlay similarity, which is calculated based on the steric and electrostatic overlaps, is high with a value of 0.86 and the r.m.s.d. of the heavy atoms (non-H atoms) is 0.67 Å. Geometrical parameters (*i.e*. bond lengths, bond angles and torsion angles) of the experimental and optimized structures are given in Table 2 ▸.

**Figure 5:**
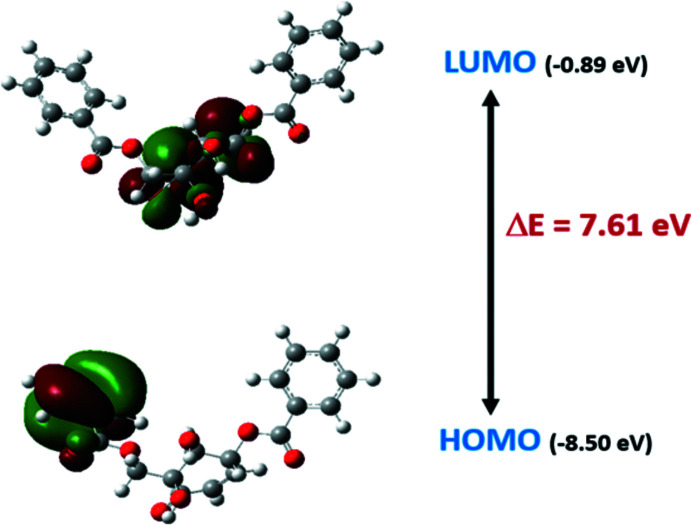
The HOMO–LUMO plot for the title compound (I).

Finally, the mol­ecular orbitals of zeylenone were calculated. The HOMO and LUMO plots are shown in Fig. 5 ▸. At the HOMO level, the orbitals are located on the phenyl ring of the methyl­ene benzoate group and the orbitals are shifted to the cyclo­hexenone ring at the LUMO level. The energy gap (*E*_HOMO_ − *E*_LUMO_) is 7.61 eV. The large energy gap indicates the stability of the title compound.

## Database survey   

In the first reported total synthesis of zeylenone from shikimic acid, the absolute configuration was assigned as 1*R*, 5*S*, 6*R*. A circular dichroism study of the synthesized product gave (+)-zeylenone (Liu *et al.*, 2004 ▸). The first total synthesis of (−)-zeylenone was also achieved from shikimic acid (Zhang *et al.*, 2006 ▸). Similar structures to (−)-zeylenone are (−)-zeylenol and an alcohol form, (−)-zeylenone, from *Piper cubeb*a (Taneja *et al.*, 1991 ▸).

The closest related structure is that of Cherrevenone, a polyoxygenated cyclo­hexene derivative from *Uvaria cherrevensis.* Here, the absolute configuration could again not be determined from the X-ray data, but was confirmed by an electronic circular dichroism analysis (CCDC refcode WOJLIT; Jaipetch *et al.*, 2019 ▸).

Other reported crystal structures containing a cyclo­hexenone ring as a core structure include URIPUH **(**Mayekar *et al.*, 2010 ▸), KADROW **(**Lynch *et al.*, 1989 ▸), WINTUI **(**Sondossi *et al.*, 1995 ▸) and CEZXUD (Atioğlu *et al.*, 2018 ▸). In all of these, the cyclo­hexenone ring adopts a half-chair conformation, as observed in the title compound.

## Synthesis and crystallization   

*Pipers griffithii* leaves, collected from Kanchanaburi province in Easten Thailand, were dried in air and then powdered with a grinder. Piper powder (400 g) was macerated at room temperature in hexane for a week and then filtered. This was repeated with the remaining *Piper* powder using ethyl acetate. The filtrate was evaporated to yield about 2.60 g crude extract from ethyl acetate, which was dissolved again in ethyl acetate and mixed with silica gel. The mixture was evaporated by rotary evaporator, loaded on the column and eluted by gradient elution using 20–50% EtOAc in hexane. The fractions were collected and combined, monitoring with thin layer chromatography, to provide eleven fractions. The sixth fraction was separated by column chromatography using MeOH:EtOAc:Hexane (1:4:5) as eluents, yielding a pale-yellow solid (0.60 g), which was recrystallized from di­chloro­methane and hexane (1:1), giving colourless in CIF crystals, m.p. 423–424 K.

^1^H NMR (400 MHz, CDCl_3_): δ 3.22 (1H, *s*, *br*), 4.11 (1H, *s*, *br*), 4.38 (1H, *d*, *J* = 4 Hz), 4.60 (1H, *d*, *J* = 12 Hz), 4.85 (1H, *d*, *J* = 8 Hz), 5.96 (1H, *d*, *J* = 4 Hz), 6.34 (1H, *dd*, *J* = 8, 8 Hz), 6.96 (1H, *dd*, *J* = 4, 8 Hz), 7.38–7.44 (4H, *m*), 7.56 (2H, *dd*, *J* = 8, 16 Hz), 7.94 (2H, *dd*, *J* = 4, 8 Hz), 8.02 (2H, *dd*, *J* = 4, 8 Hz). ^13^C NMR (CDCl_3_): δ 65.4, 69.2, 71.6, 77.2, 128.4, 128.5, 128.6, 128.7, 129.1, 129.7, 129.78, 133.4, 133.7, 142.6, 165.3, 166.1, 196.2. Mass spectroscopy *m*/*z* 383.1125 (*M* + 1)^+^. IR (KBr, cm^−1^): 712 cm^−1^ (*s*, C—H bending); 1103 cm^−1^ (*s*, C—O stretching); 1277 cm^−1^ (*s*, C—O stretching); 1593 cm^−1^ (*w*, C=C aromatic ring); 1705 cm^−1^ (*s*, C=O) ; 2933 cm^−1^ (*w*, C=C—H stretching aromatic ring); 3423 cm^−1^ (*s*, O—H stretching).

## Refinement   

Crystal data, data collection and structure refinement details are summarized in Table 3 ▸. All H atoms, ternary C(H), secondary C(H,H), aromatic H and tetra­hedral OH, were placed in calculated positions (C—H = 0.98, 0.97, 0.93 and 0.82 Å, respectively). They are refined using a riding model with *U*_iso_(H) = 1.5*U*_eq_(C) or 1.5*U*_eq_(O).

## Supplementary Material

Crystal structure: contains datablock(s) I. DOI: 10.1107/S2056989020007793/vm2234sup1.cif

Structure factors: contains datablock(s) I. DOI: 10.1107/S2056989020007793/vm2234Isup2.hkl

Supporting information file. DOI: 10.1107/S2056989020007793/vm2234Isup3.cml

CCDC reference: 2008756

Additional supporting information:  crystallographic information; 3D view; checkCIF report

## Figures and Tables

**Table 1:** Hydrogen-bond geometry (Å, °)

*D*—H⋯*A*	*D*—H	H⋯*A*	*D*⋯*A*	*D*—H⋯*A*
O2—H2⋯O7^i^	0.82	1.93 (1)	2.7029 (17)	157 (1)
O1—H1⋯O5^ii^	0.82	1.89 (1)	2.7112 (17)	177 (2)
C13—H13⋯O2^iii^	0.93 (1)	2.53 (1)	3.221 (3)	132 (1)

Symmetry codes: (i) 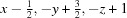; (ii) 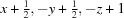; (iii) 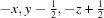.

**Table 2:** Comparison of geometric parameters (Å, °) between the experimental and optimized structures

Parameter	Exp.	Calc.	Parameter	Exp.	Calc.
O1—C1	1.425 (2)	1.43	C5—C6	1.512 (2)	1.53
O2—C6	1.403 (2)	1.40	C8—C9	1.480 (2)	1.49
O3—C2	1.211 (2)	1.21	C9—C10	1.372 (3)	1.40
O4—C7	1.446 (2)	1.43	C9—C14	1.384 (2)	1.40
O4—C8	1.327 (2)	1.35	C10—C11	1.385 (3)	1.39
O5—C8	1.196 (2)	1.21	C11—C12	1.382 (3)	1.39
O6—C5	1.455 (2)	1.43	C12—C13	1.346 (3)	1.39
O6—C15	1.334 (2)	1.35	C13—C14	1.378 (3)	1.39
O7—C15	1.206 (2)	1.21	C15—C16	1.476 (2)	1.49
C1—C2	1.534 (2)	1.54	C16—C17	1.392 (2)	1.40
C1—C6	1.530 (2)	1.53	C16—C21	1.382 (3)	1.40
C1—C7	1.509 (2)	1.52	C17—C18	1.376 (3)	1.39
C2—C3	1.456 (3)	1.47	C18—C19	1.364 (3)	1.39
C3—C4	1.322 (3)	1.34	C19—C20	1.377 (3)	1.39
C4—C5	1.489 (3)	1.50	C20—C21	1.381 (3)	1.39
					
O1—C1—C7	108.46 (13)	108.9	C2—C1—C6	108.75 (13)	113.5
O4—C8—C9	113.45 (13)	113.1	C3—C4—C5	123.74 (19)	122.6
O6—C5—C6	106.27 (11)	106.4	C4—C5—C6	112.53 (15)	111.9
O6—C15—C16	113.40 (14)	112.5	C5—O6—C15	116.55 (12)	115.8
C1—O1—H1	109.5	108.0	C6—O2—H2	109.5	106.4
C1—C2—C3	115.64 (16)	118.1	C8—O4—C7	116.34 (13)	114.4
C1—C6—C5	108.92 (12)	110.1	C8—C9—C10	122.49 (14)	112.7
C1—C7—O4	108.20 (12)	107.2	C15—C16—C21	122.2 (2)	122.19 (15)
					
O6—C15—C16—C21	−0.4 (3)	3.5	C8—O4—C7—C1	175.99 (13)	179.9
C5—O6—C15—C16	−179.95 (15)	−175.2	C9—C8—O4—C7	173.83 (14)	179.6
C6—C1—C2—C3	43.70 (19)	19.5	C10—C9—C8—O4	−11.7 (2)	3.0
C6—C5—C4—C3	−19.8 (3)	−29.8			

**Table 3:** Experimental details

Crystal data	
Chemical formula	C_21_H_18_O_7_
*M*_r_	382.37
Crystal system, space group	Orthorhombic, *P*2_1_2_1_2_1_
Temperature (K)	296
*a*, *b*, *c* (Å)	7.4958 (11), 12.422 (2), 20.325 (4)
*V* (Å^3^)	1892.4 (6)
*Z*	4
Radiation type	Mo *K*α
μ (mm^−1^)	0.10
Crystal size (mm)	0.24 × 0.08 × 0.04

Data collection	
Diffractometer	Bruker APEXII D8 QUEST CMOS
Absorption correction	Multi-scan (*SADABS*; Bruker, 2016 ▸)
*T*_min_, *T*_max_	0.708, 0.745
No. of measured, independent and observed [*I* ≥ 2u(*I*)] reflections	36963, 3587, 3199
*R*_int_	0.045
(sin θ/λ)_max_ (Å^−1^)	0.611

Refinement	
*R*[*F* ^2^ > 2σ(*F* ^2^)], *wR*(*F* ^2^), *S*	0.032, 0.082, 1.10
No. of reflections	3587
No. of parameters	255
H-atom treatment	H-atom parameters constrained
Δρ_max_, Δρ_min_ (e Å^−3^)	0.12, −0.10
Absolute structure	Flack *x* determined using 1259 quotients [(*I* ^+^)−(*I* ^−^)]/[(*I* ^+^)+(*I* ^−^)] (Parsons *et al.*, 2013 ▸)
Absolute structure parameter	0.0 (3)

Computer programs: *APEX3* and *SAINT* (Bruker, 2016 ▸), *olex2.solve* (Bourhis *et al.*, 2015 ▸), *SHELXL* (Sheldrick, 2015 ▸) and *OLEX2* (Dolomanov *et al.*, 2009 ▸).

## supplementary crystallographic information

### Crystal data

**Table:**

C_21_H_18_O_7_	*D*_x_ = 1.342 Mg m^−^^3^
*M**_r_* = 382.37	Mo *K*α radiation, λ = 0.71073 Å
Orthorhombic, *P*2_1_2_1_2_1_	Cell parameters from 9916 reflections
*a* = 7.4958 (11) Å	θ = 2.6–24.7°
*b* = 12.422 (2) Å	µ = 0.10 mm^−^^1^
*c* = 20.325 (4) Å	*T* = 296 K
*V* = 1892.4 (6) Å^3^	Plate, colourless
*Z* = 4	0.24 × 0.08 × 0.04 mm
*F*(000) = 800.5281	

### Data collection

**Table:**

Bruker APEX2 D8 QUEST CMOS diffractometer	3199 reflections with *I*≥ 2u(*I*)
ω and φ scans	*R*_int_ = 0.045
Absorption correction: multi-scan (SADABS; Bruker, 2016)	θ_max_ = 25.7°, θ_min_ = 2.9°
*T*_min_ = 0.708, *T*_max_ = 0.745	*h* = −9→9
36963 measured reflections	*k* = −15→15
3587 independent reflections	*l* = −24→24

### Refinement

**Table:**

Refinement on *F*^2^	Primary atom site location: iterative
Least-squares matrix: full	H-atom parameters constrained
*R*[*F*^2^ > 2σ(*F*^2^)] = 0.032	*w* = 1/[σ^2^(*F*_o_^2^) + (0.0343*P*)^2^ + 0.1967*P*] where *P* = (*F*_o_^2^ + 2*F*_c_^2^)/3
*wR*(*F*^2^) = 0.082	(Δ/σ)_max_ = 0.0003
*S* = 1.10	Δρ_max_ = 0.12 e Å^−^^3^
3587 reflections	Δρ_min_ = −0.10 e Å^−^^3^
255 parameters	Absolute structure: Flack *x* determined using 1259 quotients [(*I*^+^)-(*I*^-^)]/[(*I*^+^)+(*I*^-^)] (Parsons *et al.*, 2013)
0 restraints	Absolute structure parameter: 0.0 (3)
35 constraints	

### Fractional atomic coordinates and isotropic or equivalent isotropic displacement parameters (Å^2^)

**Table:**

	*x*	*y*	*z*	*U*_iso_*/*U*_eq_	
O6	0.48269 (18)	0.70917 (9)	0.38884 (5)	0.0630 (3)	
O2	0.20328 (14)	0.56656 (10)	0.43093 (5)	0.0586 (3)	
H2	0.1932 (6)	0.5895 (17)	0.4686 (4)	0.0879 (4)*	
O1	0.43371 (18)	0.46452 (9)	0.52550 (5)	0.0637 (3)	
H1	0.477 (3)	0.4158 (8)	0.54749 (17)	0.0956 (5)*	
O4	0.25100 (18)	0.33708 (9)	0.38037 (5)	0.0649 (3)	
O5	0.0902 (2)	0.19256 (12)	0.40208 (7)	0.0929 (5)	
O7	0.5956 (3)	0.82489 (11)	0.46087 (7)	0.1010 (6)	
O3	0.6515 (2)	0.30383 (12)	0.42569 (8)	0.0923 (5)	
C8	0.1399 (2)	0.25864 (12)	0.36344 (9)	0.0586 (4)	
C6	0.3807 (2)	0.53548 (12)	0.41980 (7)	0.0452 (3)	
H6	0.3924 (2)	0.51900 (12)	0.37285 (7)	0.0542 (4)*	
C15	0.5290 (3)	0.80813 (13)	0.40783 (9)	0.0655 (5)	
C1	0.4302 (2)	0.43358 (12)	0.45798 (7)	0.0511 (4)	
C2	0.6172 (3)	0.39738 (15)	0.43677 (8)	0.0639 (5)	
C16	0.4927 (2)	0.89129 (13)	0.35772 (9)	0.0605 (4)	
C10	0.1230 (2)	0.34822 (16)	0.25339 (9)	0.0660 (5)	
H10	0.1836 (2)	0.40757 (16)	0.27012 (9)	0.0793 (5)*	
C7	0.2932 (3)	0.34568 (14)	0.44959 (8)	0.0637 (4)	
H7a	0.1864 (3)	0.36290 (14)	0.47445 (8)	0.0764 (5)*	
H7b	0.3402 (3)	0.27786 (14)	0.46567 (8)	0.0764 (5)*	
C5	0.5144 (2)	0.62285 (13)	0.43577 (8)	0.0567 (4)	
H5	0.4933 (2)	0.64941 (13)	0.48050 (8)	0.0681 (5)*	
C9	0.0875 (2)	0.26165 (13)	0.29324 (8)	0.0560 (4)	
C21	0.4190 (3)	0.86669 (15)	0.29726 (9)	0.0651 (4)	
H21	0.3903 (3)	0.79577 (15)	0.28711 (9)	0.0781 (5)*	
C12	−0.0180 (3)	0.2577 (2)	0.16356 (11)	0.0823 (6)	
H12	−0.0545 (3)	0.2567 (2)	0.11984 (11)	0.0988 (7)*	
C11	0.0688 (3)	0.3472 (2)	0.18828 (10)	0.0775 (5)	
H11	0.0905 (3)	0.4063 (2)	0.16137 (10)	0.0930 (6)*	
C3	0.7495 (3)	0.4826 (2)	0.43087 (11)	0.0824 (6)	
H3	0.8696 (3)	0.4646 (2)	0.42780 (11)	0.0989 (7)*	
C4	0.7022 (3)	0.58513 (18)	0.42980 (11)	0.0776 (6)	
H4	0.7913 (3)	0.63662 (18)	0.42504 (11)	0.0931 (7)*	
C13	−0.0499 (3)	0.1720 (2)	0.20235 (13)	0.0959 (7)	
H13	−0.1063 (3)	0.1117 (2)	0.18499 (13)	0.1150 (9)*	
C19	0.4324 (3)	1.05213 (18)	0.26647 (13)	0.0881 (7)	
H19	0.4110 (3)	1.10627 (18)	0.23586 (13)	0.1057 (8)*	
C20	0.3877 (3)	0.94726 (18)	0.25191 (11)	0.0797 (6)	
H20	0.3365 (3)	0.93070 (18)	0.21149 (11)	0.0957 (7)*	
C14	0.0004 (3)	0.17290 (16)	0.26754 (11)	0.0831 (6)	
H14	−0.0243 (3)	0.11396 (16)	0.29425 (11)	0.0997 (7)*	
C18	0.5079 (4)	1.07685 (15)	0.32565 (14)	0.0927 (7)	
H18	0.5396 (4)	1.14762 (15)	0.33491 (14)	0.1112 (9)*	
C17	0.5372 (3)	0.99762 (15)	0.37173 (11)	0.0815 (6)	
H17	0.5869 (3)	1.01515 (15)	0.41227 (11)	0.0978 (7)*	

### Atomic displacement parameters (Å^2^)

**Table:**

	*U*^11^	*U*^22^	*U*^33^	*U*^12^	*U*^13^	*U*^23^
O6	0.0899 (8)	0.0477 (6)	0.0515 (6)	−0.0112 (6)	−0.0152 (6)	0.0009 (5)
O2	0.0561 (6)	0.0730 (7)	0.0468 (6)	0.0150 (6)	−0.0037 (5)	0.0023 (5)
O1	0.0901 (9)	0.0632 (7)	0.0378 (5)	0.0172 (6)	−0.0117 (5)	0.0021 (5)
O4	0.0891 (8)	0.0562 (6)	0.0493 (6)	−0.0231 (6)	−0.0050 (6)	0.0057 (5)
O5	0.1161 (12)	0.0786 (9)	0.0841 (10)	−0.0411 (9)	0.0108 (9)	0.0159 (8)
O7	0.1583 (16)	0.0694 (9)	0.0753 (9)	−0.0311 (10)	−0.0384 (10)	−0.0099 (7)
O3	0.1188 (12)	0.0758 (9)	0.0822 (9)	0.0425 (9)	0.0007 (8)	−0.0066 (8)
C8	0.0596 (9)	0.0467 (8)	0.0694 (10)	−0.0101 (7)	0.0105 (8)	−0.0016 (8)
C6	0.0513 (8)	0.0480 (7)	0.0362 (7)	0.0027 (6)	−0.0048 (6)	−0.0007 (6)
C15	0.0818 (12)	0.0530 (9)	0.0616 (10)	−0.0123 (9)	−0.0062 (9)	−0.0082 (8)
C1	0.0647 (9)	0.0509 (8)	0.0377 (7)	0.0075 (7)	−0.0067 (7)	0.0028 (6)
C2	0.0741 (11)	0.0695 (11)	0.0479 (9)	0.0199 (9)	−0.0099 (8)	0.0000 (8)
C16	0.0651 (10)	0.0499 (8)	0.0665 (10)	−0.0060 (8)	0.0097 (8)	−0.0026 (7)
C10	0.0619 (10)	0.0719 (11)	0.0643 (10)	−0.0136 (9)	0.0003 (8)	−0.0089 (9)
C7	0.0871 (12)	0.0570 (9)	0.0470 (8)	−0.0092 (9)	−0.0014 (8)	0.0097 (8)
C5	0.0712 (10)	0.0528 (8)	0.0461 (8)	−0.0060 (8)	−0.0139 (8)	0.0019 (7)
C9	0.0475 (8)	0.0561 (9)	0.0645 (10)	−0.0053 (7)	0.0034 (7)	−0.0114 (8)
C21	0.0688 (11)	0.0619 (10)	0.0646 (10)	−0.0105 (9)	0.0071 (9)	0.0044 (8)
C12	0.0619 (11)	0.1141 (17)	0.0710 (13)	0.0022 (12)	−0.0114 (9)	−0.0289 (12)
C11	0.0674 (11)	0.0993 (15)	0.0658 (11)	−0.0051 (11)	0.0001 (9)	−0.0011 (11)
C3	0.0533 (10)	0.1045 (16)	0.0894 (14)	0.0110 (11)	−0.0122 (10)	0.0037 (12)
C4	0.0609 (11)	0.0900 (15)	0.0819 (13)	−0.0174 (10)	−0.0138 (10)	0.0071 (11)
C13	0.0948 (16)	0.0893 (16)	0.1035 (17)	−0.0167 (14)	−0.0265 (14)	−0.0284 (14)
C19	0.0926 (15)	0.0704 (13)	0.1013 (17)	0.0091 (12)	0.0195 (14)	0.0237 (12)
C20	0.0843 (13)	0.0831 (14)	0.0718 (12)	−0.0052 (11)	0.0082 (10)	0.0185 (11)
C14	0.0847 (13)	0.0656 (12)	0.0991 (16)	−0.0195 (11)	−0.0116 (12)	−0.0095 (11)
C18	0.1104 (17)	0.0476 (10)	0.120 (2)	0.0003 (12)	0.0186 (16)	0.0019 (11)
C17	0.1022 (16)	0.0524 (10)	0.0900 (14)	−0.0077 (10)	0.0053 (12)	−0.0104 (9)

### Geometric parameters (Å, º)

**Table:**

O6—C15	1.3344 (19)	C7—H7a	0.9700
O6—C5	1.4545 (19)	C7—H7b	0.9700
O2—H2	0.8200	C5—H5	0.9800
O2—C6	1.4033 (18)	C5—C4	1.489 (3)
O1—H1	0.8200	C9—C14	1.384 (2)
O1—C1	1.4253 (17)	C21—H21	0.9300
O4—C8	1.3270 (19)	C21—C20	1.381 (3)
O4—C7	1.4460 (19)	C12—H12	0.9300
O5—C8	1.196 (2)	C12—C11	1.382 (3)
O7—C15	1.206 (2)	C12—C13	1.346 (3)
O3—C2	1.211 (2)	C11—H11	0.9300
C8—C9	1.480 (2)	C3—H3	0.9300
C6—H6	0.9800	C3—C4	1.322 (3)
C6—C1	1.530 (2)	C4—H4	0.9300
C6—C5	1.512 (2)	C13—H13	0.9300
C15—C16	1.476 (2)	C13—C14	1.378 (3)
C1—C2	1.534 (2)	C19—H19	0.9300
C1—C7	1.509 (2)	C19—C20	1.377 (3)
C2—C3	1.456 (3)	C19—C18	1.364 (3)
C16—C21	1.382 (3)	C20—H20	0.9300
C16—C17	1.392 (2)	C14—H14	0.9300
C10—H10	0.9300	C18—H18	0.9300
C10—C9	1.372 (3)	C18—C17	1.376 (3)
C10—C11	1.385 (3)	C17—H17	0.9300
			
C5—O6—C15	116.55 (12)	C4—C5—O6	109.45 (15)
C6—O2—H2	109.5	C4—C5—C6	112.53 (15)
C1—O1—H1	109.5	C4—C5—H5	109.51 (11)
C7—O4—C8	116.34 (13)	C10—C9—C8	122.49 (14)
O5—C8—O4	121.95 (17)	C14—C9—C8	117.97 (17)
C9—C8—O4	113.45 (13)	C14—C9—C10	119.54 (18)
C9—C8—O5	124.59 (16)	H21—C21—C16	119.96 (10)
H6—C6—O2	107.42 (7)	C20—C21—C16	120.09 (18)
C1—C6—O2	112.06 (13)	C20—C21—H21	119.96 (13)
C1—C6—H6	107.42 (8)	C11—C12—H12	119.78 (14)
C5—C6—O2	113.32 (13)	C13—C12—H12	119.78 (13)
C5—C6—H6	107.42 (8)	C13—C12—C11	120.4 (2)
C5—C6—C1	108.92 (12)	C12—C11—C10	119.5 (2)
O7—C15—O6	121.68 (16)	H11—C11—C10	120.23 (13)
C16—C15—O6	113.40 (14)	H11—C11—C12	120.23 (14)
C16—C15—O7	124.92 (16)	H3—C3—C2	119.37 (11)
C6—C1—O1	105.64 (12)	C4—C3—C2	121.27 (19)
C2—C1—O1	109.44 (13)	C4—C3—H3	119.37 (13)
C2—C1—C6	108.75 (13)	C3—C4—C5	123.74 (19)
C7—C1—O1	108.46 (13)	H4—C4—C5	118.13 (10)
C7—C1—C6	112.11 (13)	H4—C4—C3	118.13 (13)
C7—C1—C2	112.22 (14)	H13—C13—C12	119.73 (13)
C1—C2—O3	121.86 (19)	C14—C13—C12	120.5 (2)
C3—C2—O3	122.50 (19)	C14—C13—H13	119.73 (14)
C3—C2—C1	115.64 (16)	C20—C19—H19	119.90 (14)
C21—C16—C15	122.19 (15)	C18—C19—H19	119.90 (12)
C17—C16—C15	118.61 (18)	C18—C19—C20	120.2 (2)
C17—C16—C21	119.19 (18)	C19—C20—C21	120.1 (2)
C9—C10—H10	120.00 (10)	H20—C20—C21	119.96 (13)
C11—C10—H10	120.00 (13)	H20—C20—C19	119.96 (14)
C11—C10—C9	120.00 (18)	C13—C14—C9	119.9 (2)
C1—C7—O4	108.20 (12)	H14—C14—C9	120.04 (13)
H7a—C7—O4	110.06 (10)	H14—C14—C13	120.04 (14)
H7a—C7—C1	110.06 (10)	H18—C18—C19	119.82 (13)
H7b—C7—O4	110.06 (9)	C17—C18—C19	120.4 (2)
H7b—C7—C1	110.06 (9)	C17—C18—H18	119.82 (14)
H7b—C7—H7a	108.4	C18—C17—C16	120.1 (2)
C6—C5—O6	106.27 (11)	H17—C17—C16	119.96 (13)
H5—C5—O6	109.51 (8)	H17—C17—C18	119.96 (14)
H5—C5—C6	109.51 (8)		

### Hydrogen-bond geometry (Å, º)

**Table:**

*D*—H···*A*	*D*—H	H···*A*	*D*···*A*	*D*—H···*A*
O2—H2···O7^i^	0.82	1.93 (1)	2.7029 (17)	157 (1)
O1—H1···O5^ii^	0.82	1.89 (1)	2.7112 (17)	177 (2)
C13—H13···O2^iii^	0.93 (1)	2.53 (1)	3.221 (3)	132 (1)

Symmetry codes: (i) *x*−1/2, −*y*+3/2, −*z*+1; (ii) *x*+1/2, −*y*+1/2, −*z*+1; (iii) −*x*, *y*−1/2, −*z*+1/2.
